# Point-of-care testing: Connecting communities in Africa and ensuring equity in access to health and diagnostics

**DOI:** 10.4102/ajlm.v11i1.2072

**Published:** 2022-12-13

**Authors:** Rajiv T. Erasmus

**Affiliations:** 1 Department of Chemical Pathology, Faculty of Medicine and Health Sciences, Stellenbosch University, Cape Town, South Africa

According to the African Society for Laboratory Medicine, ‘[*m*]edical laboratories play a pivotal role in global disease diagnosis, surveillance, outbreak investigation, initiation and monitoring of therapy, as well as research and development’. However medical laboratories in Africa cannot meet the testing demands of a rapidly growing health delivery service,^1^ due to a lack of resources and inadequate diagnostic services, which compromise patient care. This leads to misdiagnosis, resulting in under- or over-treatment of disease, giving rise to numerous societal health challenges.^1^ A recent Lancet Commission review^2^ revealed a lack of access to diagnostics at the lowest tier of the healthcare system – the community – where the highest population in need of services exists, but only 47% globally have access. In its mapping exercise undertaken between November 2019 and April 2020, the European Centre for Disease Prevention and Control^3^ found that point-of-care (POC) testing could present a possible solution as it plays:

a critical role in rapidly diagnosing both infectious and noninfectious diseases and is not only for appropriate and timely treatment but also for the detection of outbreaks and controlling the rapid spread of infectious diseases. (p. 1)

In 2013, almost a decade ago, Jani and Peter^4^ had stressed ‘how point-of-care testing could drive innovation in Global Health’. Indeed, if one fast-forwards to 2022, POC testing has made great strides in diagnosing and managing the disease burden in Africa. According to World Health Organization,^5^ the diseases afflicting the African population are responsible for a substantial loss in health, estimated at 704 765 879 disability adjusted life years in 2015 alone. In the World Health Organization African Region, total losses amounted to 629 603 271 disability adjusted life years; 59.1% were from communicable, maternal, perinatal and nutritional conditions with diabetes, anaemia, malaria and syphilis contributing to the greatest burden of disease.^5^

The International Organization for Standardization defines POC testing and near-patient testing for any disease (not just infectious diseases) as ‘testing that is performed near or at the site of a patient with the result leading to possible change in the care of the patient’.^6^ The small footprint of the instrument means that they can be easily transported. According to the United States’ National Institutes of Health, ‘empowering clinicians to make decisions at the “point-of-care” has the potential to significantly impact healthcare delivery and to address the challenges of health disparities’ and this could result in ‘the success of a potential shift from curative medicine, to predictive, personalised, and pre-emptive medicine’.^7^ Although POC testing is progressively being used for the identification and management of various disease states in Africa, controlling various POC instruments at numerous sites can be difficult, particularly when such instruments are run by non-technical staff. However, these challenges can be overcome with recent advances in connectivity and digitisation, as well as standardisation of software and middleware, which allow the quality of testing to be monitored in real time.

Connectivity allows for POC instruments to hook up with a laboratory or hospital information system, as well as with electronic medical records.^8^ For this purpose an open-access data management system is critical, permitting connections between instruments from any manufacturer. This process automatically validates and transfers patient results, including those from electronic medical records, as well as monitoring and managing data, multiple testing devices, and operators.^8^ As these are intimately linked to operator training and competency, harm to the patient is minimised by reducing analytical errors. Faster decision-making can also occur due to significantly lower turnaround times.^8^ With the advent of 5G and artificial intelligence, greater focus on personalised care is likely to occur resulting from big data analysis and development of algorithms; communities in Africa stand to benefit from this. Wireless technology, the Internet of Things and big data will not only allow patients in Africa to take charge of their health needs, but hugely improve the way health-related data is transmitted and interpreted, making it possible for healthcare delivery to be more efficient, precise and, ultimately, more affordable. In addition, accessibility would mean increased equity in healthcare and diagnostics to communities in Africa. Smartphone-based operation, paper-based sensing assays, and lab-on-a-chip are being turned into mobile laboratories, which are furthering this trend in remote areas.^9^

Already there are well-established programmes that have changed the health landscape in Africa. The most recent Cochrane review^10^ reported that POC viral load testing showed both high sensitivity and specificity for the diagnosis of HIV viral load, at or above the clinical threshold of 1000 copies/mL, among people living with HIV who attended healthcare facilities for their HIV tests. Furthermore, POC viral load testing has been reported to enhance viral suppression and retention in HIV care.^10,11^ Rapid antigen lateral flow tests were particularly useful during the coronavirus disease 2019 (COVID-19) pandemic and were used for rapid screening and tracing of cases in communities.^12^ The introduction of POC lateral flow urine lipoarabinomannan assays has improved tuberculosis case detection and lowered diagnostic holdups in people living with HIV and is particularly useful in African communities where traditional methods have a long turnaround time.

Although education, training, data governance and quality management will remain key to ensure the reliability and accuracy of test results,^13,14^ these results demonstrate that POC testing can simplify treatment and improve outcomes for HIV-positive adults receiving antiretroviral therapy in resource-limited settings and that POC testing presents a great advantage for disease diagnosis, monitoring and its management for patients in Africa. The African Union^11^ has put forward a digital transformation strategy for Africa 2020–2030. If it is successfully implemented, it will no doubt further propel POC testing, ensuring optimal governance and quality. Thus, Africa is in a unique position to use POC and digital technologies, including machine learning and artificial intelligence, into a highly effective strategy for delivering speedy, high-quality, data-driven care to communities in Africa. Africa must take advantage of the convergence of POC testing and digital technologies, as this will surely enhance diagnostics and healthcare on the continent.
